# Next-Generation Sequencing Approach in Methylation Analysis of *HNF1B* and *GATA4* Genes: Searching for Biomarkers in Ovarian Cancer

**DOI:** 10.3390/ijms18020474

**Published:** 2017-02-22

**Authors:** Ivana Bubancova, Helena Kovarikova, Jan Laco, Ema Ruszova, Ondrej Dvorak, Vladimir Palicka, Marcela Chmelarova

**Affiliations:** 1 Institute of Clinical Biochemistry and Diagnostics, Charles University Faculty of Medicine and University Hospital Hradec Kralove, 500 05 Hradec Kralove, Czech Republic; kovarikovahe@lfhk.cuni.cz (H.K.); palicka@lfhk.cuni.cz (V.P.); marcela.chmelarova@fnhk.cz (M.C.); 2 The Fingerland Department of Pathology, Charles University Faculty of Medicine and University Hospital Hradec Kralove, 500 05 Hradec Kralove, Czech Republic; lacoj@lfhk.cuni.cz; 3 Department of Clinical Genetics, University Hospital Hradec Kralove, 500 05 Hradec Kralove, Czech Republic; ema.ruszova@fnhk.cz; 4 Department of Obstetrics and Gynecology, Charles University Faculty of Medicine and University Hospital Hradec Kralove, 500 05 Hradec Kralove, Czech Republic; ondrej.dvorak@fnhk.cz

**Keywords:** ovarian cancer, methylation, *GATA4*, *HNF1B*, next-generation sequencing, biomarker

## Abstract

DNA methylation is well-known to be associated with ovarian cancer (OC) and has great potential to serve as a biomarker in monitoring response to therapy and for disease screening. The purpose of this study was to investigate methylation of *HNF1B* and *GATA4* and correlate detected methylation with clinicopathological characteristic of OC patients. The study group consisted of 64 patients with OC and 35 control patients. To determine the most important sites of *HNF1B* and *GATA4*, we used next-generation sequencing. For further confirmation of detected methylation of selected regions, we used high-resolution melting analysis and methylation-specific real-time polymerase chain reaction (PCR). Selected regions of *HNF1B* and *GATA4* were completely methylation free in all control samples, whereas methylation-positive pattern was observed in 32.8% (*HNF1B*) and 45.3% (*GATA4*) of OC samples. Evaluating both genes together, we were able to detect methylation in 65.6% of OC patients. We observed a statistically significant difference in *HNF1B* methylation between samples with different stages of OC. We also detected subtype specific methylation in *GATA4* and a decrease of methylation in late stages of OC. The combination of unmethylated *HNF1B* and methylated *GATA4* was associated with longer overall survival. In our study, we employed innovative approach of methylation analysis of *HNF1B* and *GATA4* to search for possible epigenetic biomarkers. We confirmed the significance of the *HNF1B* and *GATA4* hypermethylation with emphasis on the need of selecting the most relevant sites for analysis. We suggest selected CpGs to be further examined as a potential positive prognostic factor.

## 1. Introduction

Ovarian cancer (OC) includes several types of cancer that arise from different cells within the ovary. The most common type is epithelial OC, a heterogenous cancer with five major histologically distinct subtypes: high-grade serous, low-grade serous, endometrioid, clear cell, and mucinous [1]. Due to the lack of any specific symptoms in early stages, most cases of OC are diagnosed at an advanced stage after the disease has spread beyond the ovary. The aggressive nature of the disease, in addition to the lack of effective screening test, causes OC to have the worst mortality rate of all female reproductive system cancers. In 2012, there were estimated 65,540 new cases of OC in Europe, giving an incidence rate of 13.1/100,000 women, and 42,716 deaths, giving a mortality rate of 7.6/100,000 women [2].

While the molecular mechanisms of OC remain unclear, many different factors may contribute to OC development. Progressive genetic alterations (mutations involving oncogenes or tumor suppressor genes, as well as chromosomal abnormalities), hormonal profile (high number of ovulatory cycles due to early onset of menses or late menopause, infertility, endometriosis), age and lifestyle factors (diet, obesity, smoking), geographical areas, and racial and ethnic variation are all involved in cancerogenesis [3]. More recently, it has been demonstrated that OC is also driven by epigenetic alterations [4]. The most commonly-occurring epigenetic event taking place in the mammalian genome is DNA methylation, the addition of a methyl group to the 5′-carbon of cytosine in CpG sequences. CpG rich regions located in the 5′ region of genes are associated with their promoters and the regulation of gene transcription [5]. DNA methylation, though heritable, is reversible, making it a potential therapeutic target. It has been suggested that epigenetic alterations have a great potential to serve as biomarkers in monitoring response to therapy and for disease screening and detection [6]. Much effort is thus devoted to detecting suitable genes for this role. Many different genes have been identified as being hypermethylated and silenced in ovarian carcinoma [7,8].

It is well known that transcription factors can dominantly act as oncogenes or tumor suppressor genes. Some of transcription factors are not conventional tumor suppressors, but their loss contributes to malignant transformation. They normally promote cellular differentiation of numerous tissues and therefore their impaired function or altered expression may contribute to the malignant transformation of affected cells [9].

In this study, we used next-generation sequencing (NGS) for detecting regions with the most altered methylation status of transcription factors HNF1B and GATA4 in OC tissue in comparison with normal tissue. To confirm discovered alteration, we further analyzed ovarian tissue from more extensive group of patients using high-resolution melting analysis (HRM) and methylation-specific real-time PCR (MS-PCR).

## 2. Results

### 2.1. Next-Generation Sequencing

**Figure 1:**
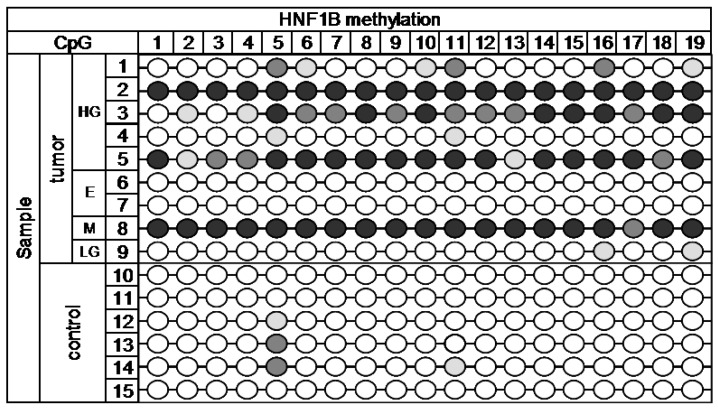
Next-generation sequencing (NGS) methylation data of *HNF1B*. Methylation: 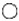 < 15%, 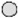 15%–24.99%, 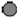 25%–49.99%, 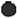 ˃ 50%. Histology: HG—high-grade serous, E—endometrioid, M—mucinous, LG—low-grade serous.

**Figure 2:**
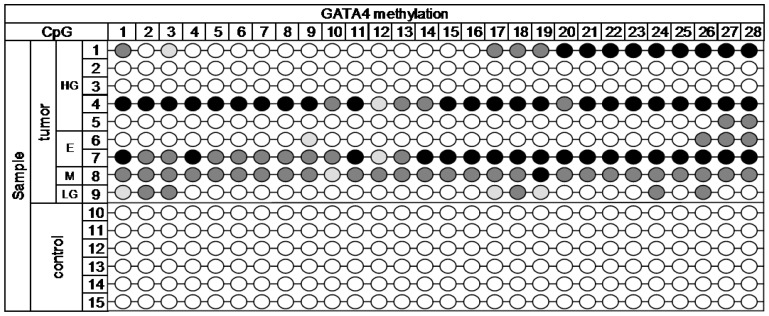
NGS methylation data of *GATA4.* Methylation: 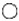 < 15%, 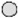 15%–24.99%, 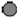 25%–49.99%, 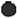 ˃ 50%. Histology: HG—high-grade serous, E—endometrioid, M—mucinous, LG—low-grade serous.

**Figure A1:**
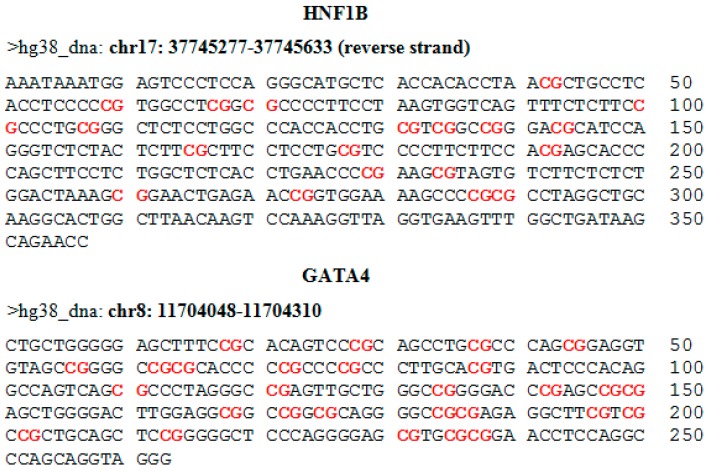
Genomic coordinates and analyzed CpG sites in *HNF1B* and *GATA4*.

For the evaluation of methylation using NGS, we selected 19 target loci in the promoter region of the *HNF1B* gene and 28 target loci in the promoter and first exon of the *GATA4* gene. Genomic coordinates and sequences with highlighted CpG dinucleotides are shown in the appendix in Figure A1. The MiSeq sequencing run produced 11.43 million reads, with 10.68 million passing filter and an average number of reads per sample of 1700. The library was spiked with 20% PhiX control, and 9.8% of total reads were aligned to the PhiX genome. The DNA methylation profile in nine cancerous and six control samples is depicted in Figure 1 (*HNF1B* gene) and Figure 2 (*GATA4* gene).

Statistically significant methylation (*p* = 0.004) was present across the 19 analyzed target CpG loci of *HNF1B* gene. In CpG5, 6, 10, 11, 16, and 19 there was high methylation present in over 50% of tumor samples; the rest of CpGs was methylated at least in 30% of tumor samples. Only minor methylation was present in two CpGs (5 and 11) in some control samples. We avoided these sites in the selection of regions for further analysis by HRM.

Across the 28 analyzed target CpG loci of *GATA4* gene, statistically significant methylation (*p* < 0.001) was detected. High methylation was present in over 50% of tumor samples in CpG1, 3, 17, 18, 19, 24, 26, 27, and 28. The rest of CpGs was methylated in at least 30% of the tumor samples. The selected region of *GATA4* gene was completely methylation free in all of the control samples.

### 2.2. High-Resolution Melting Analysis of HNF1B Gene

Based on the results from NGS, we selected a region of the *HNF1B* gene for further analysis by HRM. The analyzed region covers CpGs 16–19, where methylation was detected in over 60% of tumor samples and none of control samples. To confirm hypermethylation in the *HNF1B* gene, we analyzed 124 samples (64 cancer samples and 60 control samples). *HNF1B* in all of the control samples was completely methylation free. A statistically significant (*p* < 0.001) methylation-positive pattern was observed in 32.8% (21/64) of OC tissue samples. The lower presence of methylation in comparison with results from NGS is due to the assay design. Based on results from samples that underwent both analyses, NGS and HRM, a sample is shown as definitely positive only if every CpG site in the amplicon is methylated. In case there is one or more unmethylated CpGs, methylation status is influenced by the ratio between CpGs that are considered unmethylated and methylated. The correlations of detected methylation with clinicopathological characteristics including age, tumor stage, and histological type are summarized in Table 1. In the late stage tumors, methylation was detected in 47.4% (18/38) of cases, whereas in the early stage tumors it was only detected in 11.5% (3/26). This is considered a very significant statistical difference (*p* = 0.003). No significant correlation between *HNF1B* methylation and age or histological type was observed.

### 2.3. Real-Time Methylation Specific Analysis of GATA4 Gene

To confirm the presence of methylation detected by NGS in CpGs 22–25 of selected region of *GATA4* gene we further analyzed 124 samples (64 cancer samples and 60 control samples). Statistically significant (*p* < 0.001) methylation-positive pattern was observed in 45.3% (29/64) of OC tissue samples. All control samples were methylation free. The correlations of detected methylation with clinicopathological characteristics including age, tumor stage, and histological type are summarized in Table 1. Borderline significant correlation (*p* = 0.05) was found between various histology types, specifically between endometrioid and high-grade serous types. In the endometrioid type tumors, methylation was present in 69.2% of cases (9/13), whereas methylation in high-grade serous type tumors was detected in 35% (14/40). No significant correlation between *GATA4* methylation and age or stage was observed.

### 2.4. Follow-Up

During the follow-up period, until September 2016, relapses occurred in 24/57 (42.1%) of patients and 28/57 (49.1%) patients died due to OC. We were unable to obtain follow-up data of seven patients, as they were subsequently treated in another hospital. Overall survival of patients ranged from 2–189 months, with a median of 64 months.

**Figure 3:**
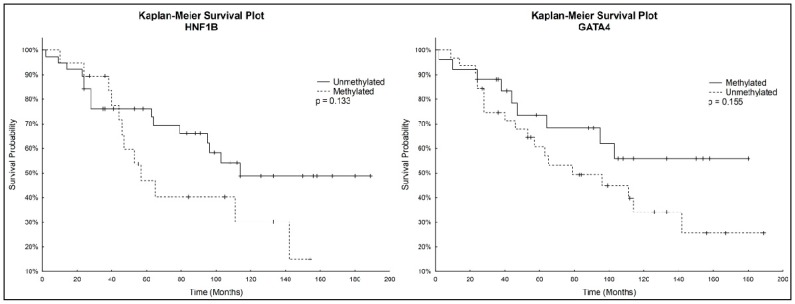
Survival probability of patients with methylated or unmethylated *HNF1B* and *GATA4*.

Kaplan-Meier analysis with the Logrank test was used to determine overall survival. Patients with unmethylated *HNF1B* had a higher survival rate than patients with methylated *HNF1B*. On the other hand, Kaplan–Meier curves showed a lower survival rate for patients with unmethylated *GATA4* compared to those with methylated *GATA4*. These correlations were not considered statistically significant. Kaplan-Meier survival plots for both genes individually are depicted in Figure 3.

**Figure 4:**
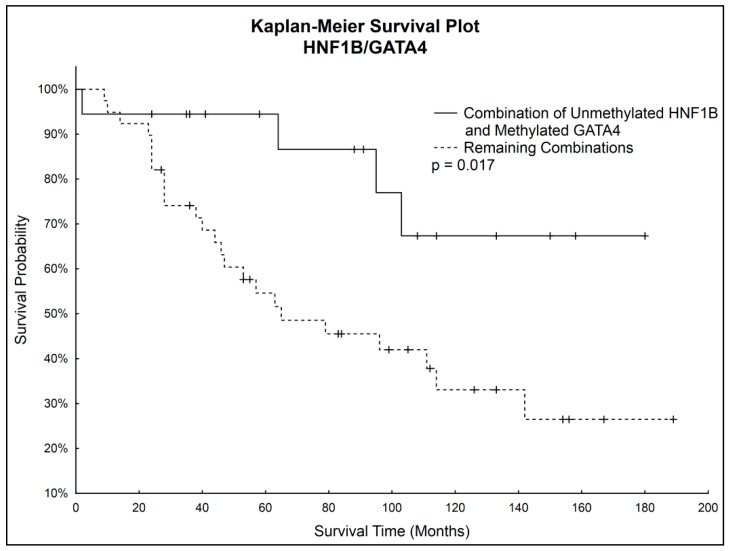
Survival probability of patients with simultaneously unmethylated *HNF1B* and methylated *GATA4*.

Simultaneous Kaplan–Meier analysis of both genes together showed statistically significantly higher survival probability (*p* = 0.017) of patients with unmethylated *HNF1B* and simultaneously methylated *GATA4* compared to the rest of the patients. Survival curves are shown in Figure 4.

No significant correlation between *HNF1B* or *GATA4* methylation and recurrence was observed.

## 3. Discussion

Epigenetic alterations, such as DNA methylation, are well-known to be involved in OC initiation and progression [10,11,12]. Aberrant DNA hypermethylation of specific genes, including genes encoding transcription factors, may result in abnormal expression in normal cells, leading to the proliferation and differentiation of tumor cells. In our study, we analyzed methylation pattern of selected regions of two genes associated with OC, specifically *HNF1B* and *GATA4*, with the aim of determining whether they can serve as potential epigenetic markers of clinical benefit in disease screening, diagnosis, and prognosis. Next-generation sequencing approach can be an invaluable tool in looking for such epigenetic biomarkers. While commonly used methods for analysis of DNA methylation can monitor only a few CpGs, NGS can provide a comprehensive view of methylation patterns of more extensive region of selected gene. Since methylation is site specific, as is evident from Figure 1 and Figure 2, it is practical to use NGS for a preliminary scan, and then for further analysis employ another method with focus on the most interesting sites of selected gene.

*HNF1B* (hepatocyte nuclear factor-1-β), also known as TCF2 (transcription factor-2), encodes a member of the homeodomain-containing superfamily of transcription factors. It is essential in the liver-specific expression of many genes during differentiation and development. Mutations in this gene result in renal cysts, diabetes syndrome, and noninsulin-dependent diabetes mellitus, and its expression is altered in several types of cancer, such as prostate cancer, endometrial cancer, or OC [13,14,15,16]. It was demonstrated by Kao et al. [17] that the overexpression of *HNF1B* is specific for clear-cell OC among ovarian carcinomas which led to its use as diagnostic marker. Epigenetic silencing of the *HNF1B* gene has also been reported in some human cancers, including colorectal carcinoma, breast cancer, or OC [18,19,20]. In our experiments we focused on DNA methylation analysis of the *HNF1B* gene. Selected region was completely methylation free in all of the control samples, whereas methylation-positive pattern was observed in 32.8% (21/64) of OC tissue samples. All tumor samples with mucinous and low-grade serous histology were methylation free, but due to the small number of these samples it is impossible to draw relevant conclusion. In the subgroup with high-grade serous tumors, methylation was detected in 45% (18/40) of samples. Our results are in correlation with previous studies [20,21] showing a slightly higher percentage of methylation that is presumably caused by our choice of more important site for analysis thanks to the initial NGS scan. In addition, we observed a statistically very significant difference (*p* = 0.003) between samples with different stages of OC. In the tumor samples with late stages was methylation present in 47.4% (18/38) of cases, whereas methylation in the samples with early stage was detected only in 11.5% (3/26).

The *GATA4* gene encodes a member of the GATA family of zinc-finger transcription factors, which recognizes GATA motif, present in the promoters of many genes. It is involved in embryogenesis and myocardial differentiation and function, and it is necessary for normal testicular development. Mutations in this gene have been associated with wide variety of congenital cardiovascular abnormalities. Loss of expression and epigenetic silencing of the *GATA4* gene has been reported in numerous types of cancer, including epithelial OC [22,23,24]. Wakana et al. [24] proved the importance of *GATA4* methylation in OC cell lines, and Montavon et al. [25] confirmed the significance of promoter methylation of the *GATA4* gene in high-grade serous OC. They found promoter methylation of the *GATA4* gene in 76.9% of OC samples and in 33.3% of benign ovarian surface epithelium samples. Although in our study methylation of the *GATA4* gene was observed only in 45.3% (29/64) of OC tissue samples, there was no methylation present in the control samples (*p* < 0.001). Another study analyzed different site in promoter region in the *GATA4* gene and found methylation present in 31.3% of OC tissue samples and in none of the samples from control group [26]. This difference in results emphasizes the need of selecting the most relevant site for analysis. Our results also showed borderline significant correlation (*p* = 0.05) between various histology types, specifically endometrioid and high-grade serous types. In the endometrioid type tumors, methylation was present in 69.2% of cases (9/13), whereas methylation in high-grade serous type ones was detected in 35% (14/40). We also observed a decrease of *GATA4* methylation in the late stage tumors compared to the early stage ones (from 53.8% to 39.5%), which shows that analyzed CpG site in the *GATA4* gene could be a promising target for early detection of OC, especially if detected in plasma.

Methylation frequency of *HNF1B* and *GATA4* in OC patients examined individually was 32.8% (21/64), resp. 45.3% (29/64). However, evaluating both genes together, we were able to detect methylation in 65.6% (42/64) of OC patients. This increase in detected methylation shows the potential of selected gene regions to be included into a DNA methylation biomarker panel.

Although correlations between methylation status of *HNF1B* or *GATA4* and overall survival were not considered statistically significant, we noticed that patients with unmethylated *GATA4* had worse survival prognosis, which correlated with observed decrease of *GATA4* methylation in the late stages of OC. Considering also the statistically significantly higher *HNF1B* methylation in the late stages of OC, we focused on further analysis of both genes combinations. A statistically significantly higher survival rate (*p* = 0.017) was found in patients with combination of unmethylated *HNF1B* and methylated *GATA4*, which shows its potential to serve as positive prognostic factor.

## 4. Materials and Methods

### 4.1. Study Group

The study group consisted of 64 patients with OC and 35 patients with a non-malignant diagnosis (such as descent of the uterus with adnexectomy or uterine leiomyomas, etc.) surgically treated at the Department of Obstetrics and Gynecology, University Hospital Hradec Kralove between years 2001–2014. According to the müllerian system theory, serous and endometrioid ovarian cancers are derived from fallopian tube and endometrium; mucinous tumors do not display a müllerian phenotype and the origin of these tumors is not entirely clear [27]. The group of control samples (obtained from 35 patients with non-malignant diagnosis, *n* = 60) therefore consisted, besides whole normal ovary samples (*n* = 35), of tissue samples from fimbriated end of fallopian tube (*n* = 15) and endometrioid tissue samples (*n* = 10). Samples from all patients were formalin-fixed, paraffin-embedded (FFPE), and fresh frozen samples were also obtained from 15 patients (nine tumors of various histology and six control samples). Samples reviewed by an experienced pathologist (J.L.) were retrieved from the archive of the Fingerland Department of Pathology, University Hospital Hradec Kralove. The study was conducted in accordance with the Declaration of Helsinki, and the protocol was approved by the Ethics Committee of University Hospital Hradec Kralove (201511 S08P). Informed consent related to fresh frozen tissue samples was obtained from each concerned patient. The need for informed consent related to FFPE tissue samples was waived by the review board in view of the retrospective nature of the study and long archival period of the samples. The carcinomas were classified according to the current WHO classification of tumors of the female genital organs [1]. Clinicopathological data of patients with OC are summarized in Table 2. The median age of patients with carcinoma at the time of diagnosis was 56 years (35–83 years), median age at the time of surgery in control group was 57 years (40–84 years).

### 4.2. DNA Extraction and Bisulfite Conversion

Genomic DNA from tissue samples was extracted using the QIAmp DNA mini kit (Qiagen, Hilden, Germany) following the manufacturer’s instruction. The purity of extracted DNA was examined spectrophotometrically and then quantified using the Qubit^®^ Flourometer (Thermo Fisher Scientific, Waltham, MA, USA). Bisulfite treatment of genomic DNA was used for conversion of all unmethylated cytosines to uracils, while leaving methylated cytosines unaffected. 500 ng of genomic DNA was treated with bisulfite using the EZ DNA Methylation-Gold™ Kit according to the manufacturer’s protocol (Zymo Research Corporation, Irvine, CA, USA).

### 4.3. Next-Generation Sequencing

NGS was performed on the MiSeq System (Illumina, San Diego, CA, USA) which uses reversible-terminator sequencing by synthesis technology capable of producing a massive parallel sequencing environment. This approach enables base-by-base sequencing with highly accurate data and together with possibility of diverse library preparation methods offers a variety of sequencing applications.

To identify regions of interest for further examination, we sequenced selected sites of a promoter region and an adjacent exon of the *HNF1B* and *GATA4* genes in 15 samples from fresh frozen ovarian tissue: nine tumors (5 high-grade serous, 2 endometrioid, 1 mucinous, and 1 low-grade serous) and six control samples. Specific primers for amplifying the regions of our interest were designed in MethPrimer. Primer sequences and amplicons information are listed in Table 3. Sequencing libraries were prepared using the Multiplicom approach. First PCR was carried out in a final volume of 20 µL containing 2 µL 10× Reaction Buffer no MgCl_2_, 2 µL MgCl_2_ (25 mM), 1.6 µL dNTPs solution Takara (2.5 mM), 1 µL of each primer (10 µM), 0.25 µL AmpliTaq Gold^®^ DNA Polymerase (Thermo Fisher Scientific), 2 µL bisulfite converted DNA, and water. All PCR amplifications were performed in the Veriti™ Thermal Cycler (Thermo Fisher Scientific). The cycling condition consisted of an initial denaturation at 95 °C for 5 min, 40 cycles of denaturing at 95 °C for 20 s, annealing at 60 °C for 30 s, and extension at 72 °C for 35 s, followed by final extension for 5 min at 72 °C. Bisulfite treated universal methylated and unmethylated DNA (Zymo Research Corporation) were used as controls. PCR products, inspected under ultraviolet light after gel electrophoresis with ethidium bromide staining, were 100× diluted and amplified in a subsequent barcoding PCR. Unique DNA sequencing barcodes and specific adapters for Illumina sequencing were incorporated into each sample using MID for the Illumina MiSeq^®^ kit (Multiplicom, Niel, Belgium) with minor modifications. PCR products were separated on 2% agarose gel and specific products were cut from gel and purified using the NucleoSpin^®^ Gel and PCR Clean-up (Macherey-Nagel, Düren, Germany). Purified sample concentrations were measured using the DQ300 Fluorometer (Hoefer, Holliston, MA, USA). All samples were equimolarly pooled into one library, which was then quantified using the KAPA library quantification assay (Kapa Biosystems, Wilmington, MA, USA) and a 4 nM library was prepared. Fragment lengths were determined by Agilent high sensitivity DNA assay using 2100 Bioanalyzer (Agilent Technologies, Santa Clara, CA, USA). NGS was carried out on Illumina MiSeq using Reagent Kit v2 at a 2 × 250 base pair read length configuration with paired-end reads following the manufacturer’s instructions. Further analysis of sequences acquired as FASTQ files and calculation of methylation status of analyzed CpG sites was performed in NextGENe^®^ software (Softgenetics, State College, PA, USA). Bisulfite-converted methylated DNA was used as a reference sequence.

### 4.4. High-Resolution Melting Analysis of HNF1B Gene

HRM analysis is an innovative technique based on analysis of melt curves of DNA following PCR amplification. A melt curve is generated by slowly denaturing the DNA sample through a range of temperatures in the presence of a double-stranded DNA binding dye. When the double-stranded DNA melts into its single-stranded form, the dye is gradually released, causing change in fluorescence, which is continuously detected by an optical system. The ability of HRM analysis to discriminate differences in base pairing enables its use for examining methylation status of bisulfite-converted DNA.

To confirm hypermethylation of selected region in the *HNF1B* gene, we analyzed 124 FFPE samples. Primers were designed using MethPrimer, considering the fact that FFPE DNA is highly fragmented and amplicons over 200 bp in length result in lower melting resolution. Sequence of forward primer was 5′-TTTTGGATTAAAGYGGAATTGAG-3′, sequence of reverse primer 5′-TCCATTATACTCACRCTAAAAAAC-3′, with amplicon length 153 bp. Amplicon included 5 CpG sites. PCR amplification and HRM analysis were performed on Rotor Gene Q (Qiagen). PCR was carried out in a final volume of 20 µL containing: 2 µL 10× Reaction Buffer no MgCl_2_, 2 µL MgCl_2_ (25 mM), 1.6 µL dNTPs solution Takara (2.5 mM), 0.5 µL of each primer (10 µM), 0.3 µl SYTO^®^ 9 Dye (0.05 mM), 0.25 µL AmpliTaq Gold^®^ DNA Polymerase (Thermo Fisher Scientific), 2 µL bisulfite converted DNA, and water. The cycling conditions were as follows: an initial denaturation step 95 °C for 5 min, 45 cycles of 95 °C for 20 s, 60 °C for 30 s, and 72 °C for 35 s, with final extension for 5 min at 72 °C, followed by a HRM step consisting of ramping between 65–85 °C by 0.1 °C with a hold of 2 s at each step. Each run included a no template control, a bisulfite-converted universal methylated and unmethylated DNA (Qiagen) and prepared standard containing 10% of universal methylated DNA which served as a cut-off for methylation status. HRM data were analyzed using Rotor gene Q software 2.3 (Qiagen).

### 4.5. Real-Time Methylation Specific Analysis of GATA4 Gene

MS-PCR assay for determining DNA methylation was used to analyze 4 selected CpGs in the *GATA4* gene. To confirm hypermethylation of selected region we analyzed 124 FFPE samples. Primers were designed using MethPrimer again with consideration of the FFPE DNA fragmentation. MS-PCR was performed on the Rotor-Gene Q (Qiagen) in two types of reaction mixture within one run, for amplifying methylated and unmethylated DNA, respectively. Primer sequences for methylated DNA were as follows: forward primer 5′-GTTTCGTCGTCGTTGTAGTTTC-3′, reverse primer 5′-ATAAAATAAATAACGCACGTCTCTT-3′, with amplicon length 197 bp. Primer sequences for unmethylated DNA were as follows: forward primer 5′-TTTGTTGTTGTTGTAGTTTTGGG-3′, reverse primer 5′-TAAAATAAATAACACACATCTCTT-3′, with amplicon length 194 bp. MS-PCR was carried out in a final volume of 20 µL containing: 2 µL 10× Reaction Buffer no MgCl_2_, 2 µL MgCl_2_ (25 mM), 1.6 µL dNTPs solution Takara (2.5 mM), 0.5 µL of each primer (10 µM), 0.3 µL SYTO^®^ 9 Dye (0.05 mM), 0.25 µL AmpliTaq Gold^®^ DNA Polymerase (Thermo Fisher Scientific), 1.5 µL bisulfite converted DNA, and water. The cycling conditions were as follows: an initial denaturation step 95 °C for 5 min, 40 cycles of 95 °C for 20 s, 58 °C for 30 s and 70 °C for 35 s. Each run included a bisulfite-converted universal methylated and unmethylated DNA (Qiagen) and a no template control. Fluorescence data were analyzed using Rotor gene Q software. The methylation status of amplicon was determined according to methylation index $M(\%)=100/\lfloor 1+2^{\left(C_{Tm}-C_{Tu}\right)}\rfloor$ (*C*_Tm_: *C*_t_ value of reaction with primer pair for methylated DNA; *C*_Tu_: *C*_t_ value of reaction with primer pair for unmethylated DNA) with cut-off 5%. When there was no reaction in reaction mixture with primer pair for methylated DNA the amplicon was considered as unmethylated.

### 4.6. Statistical Analysis

Categorical variables were compared by two-tailed Fisher’s exact test and/or Chi square test. *p*-Values < 0.05 were considered statistically significant. The Kaplan Maier method and Logrank test were used to determine overall survival rate and significance. All statistical analyses were performed using STATISTICA Cz (data analysis software system) version 12 (StatSoft, Inc., Tulsa, OK, USA).

## 5. Conclusions

In our study we employed innovative approach of selecting specific sites of the *HNF1B* and *GATA4* genes for methylation analysis in searching of possible epigenetic biomarkers. We confirmed significance of the *HNF1B* and *GATA4* hypermethylation with emphasis on the need of selecting the most relevant sites for analysis. Our results showed the presence of methylation in cancerous samples, whereas there was no methylation in the control samples. We observed a statistically very significant difference in methylation of a selected region of the *HNF1B* gene between samples with different stages of OC. We also detected subtype specific methylation in the *GATA4* gene and variances between percentages of methylation in different stages of OC. The combination of unmethylated *HNF1B* and methylated *GATA4* was associated with longer overall survival. Such epigenetic characteristics as these suggest possible use for OC screening and show potential to serve as prognostic factor.

## Figures and Tables

**Table 1:** Methylation of genes and clinicopathological characteristics.

Characteristic		*HNF1B* Methylation			*GATA4* Methylation		
		Unmet	Met	% Met	Unmet	Met	% Met
Age	≤45 years (*n* = 7)	6	1	14.3	3	4	57.1
	>45 years (*n* = 57)	37	20	35.1	32	25	43.9
Stage	I. + II. (*n* = 26)	23	3	11.5	12	14	53.8
	III. + IV. (*n* = 38)	20	18	47.4 *	23	15	39.5
Histology	high-grade serous (*n* = 40)	22	18	45.0	26	14	35.0
	endometrioid (*n* = 13)	10	3	23.1	4	9	69.2 **
	mucinous (*n* = 6)	6	0	0	2	4	66.7
	low-grade serous (*n* = 5)	5	0	0	2	3	60.0

Abbreviations: Unmet—unmethylated, Met—methylated; * *p* = 0.003, ** *p* = 0.05.

**Table 2:** Clinicopathological data of patients with ovarian cancer (OC).

Characteristic		Quantity	
		Overall	%
Age	≤45 years	7	10.9
	>45 years	57	89.1
Stage	I. + II.	26	40.6
	III. + IV.	38	59.4
Histology	high-grade serous	40	62.5
	endometrioid	13	20.3
	mucinous	6	9.4
	low-grade serous	5	7.8

**Table 3:** NGS primer sequences and amplicons information.

Gene	Primer Sequence 5′–3′ (with Adapters *)	Amplicon Size (bp) without Adapters and Barcodes	CpGs/Amplicon	Annealing Temperature (°C)
*HNF1B*	Fw: AAGACTCGGCAGCATCTCCAAAATAAATGGAGTTTTTTTAGGGTATGT Rv: GCGATCGTCACTGTTCTCCAAATTCTACTTATCAACCAAACTTCACC	357	19	60
*GATA4*	Fw: AAGACTCGGCAGCATCTCCAGATTTTGTTTGTTGGGGGAG Rv: GCGATCGTCACTGTTCTCCACCCTACCTACTAAACCTAAAAATTCC	271	28	60

* adapter overhangs: Fw: AAGACTCGGCAGCATCTCCA, Rv: GCGATCGTCACTGTTCTCCA.
